# Smart Mesoporous Nanomaterials for Antitumor Therapy

**DOI:** 10.3390/nano5041906

**Published:** 2015-11-06

**Authors:** Marina Martínez-Carmona, Montserrat Colilla, Maria Vallet-Regí

**Affiliations:** 1 Department of Inorganic and Bioinorganic Chemistry, Faculty of Pharmacy, Complutense University of Madrid, Sanitary Research Institute “Hospital 12 de Octubre” i+12, Ramón y Cajal Square, S/N, Madrid 28040, Spain; E-Mail: marinm11@ucm.es; 2 Center on Bioengineering, Biomaterials and Nanomedicine (CIBER-BBN), Madrid 28040, Spain; 3 Campus of International Excellence, CEI Campus Moncloa, UCM-UPM, Madrid 28040, Spain

**Keywords:** mesoporous silica nanoparticles, cancer treatment, passive targeting, active targeting, stimuli-responsive drug delivery

## Abstract

The use of nanomaterials for the treatment of solid tumours is receiving increasing attention by the scientific community. Among them, mesoporous silica nanoparticles (MSNs) exhibit unique features that make them suitable nanocarriers to host, transport and protect drug molecules until the target is reached. It is possible to incorporate different targeting ligands to the outermost surface of MSNs to selectively drive the drugs to the tumour tissues. To prevent the premature release of the cargo entrapped in the mesopores, it is feasible to cap the pore entrances using stimuli-responsive nanogates. Therefore, upon exposure to internal (pH, enzymes, glutathione, *etc.*) or external (temperature, light, magnetic field, *etc.*) stimuli, the pore opening takes place and the release of the entrapped cargo occurs. These smart MSNs are capable of selectively reaching and accumulating at the target tissue and releasing the entrapped drug in a specific and controlled fashion, constituting a promising alternative to conventional chemotherapy, which is typically associated with undesired side effects. In this review, we overview the recent advances reported by the scientific community in developing MSNs for antitumor therapy. We highlight the possibility to design multifunctional nanosystems using different therapeutic approaches aimed at increasing the efficacy of the antitumor treatment.

## 1. Introduction

**Figure 1:**
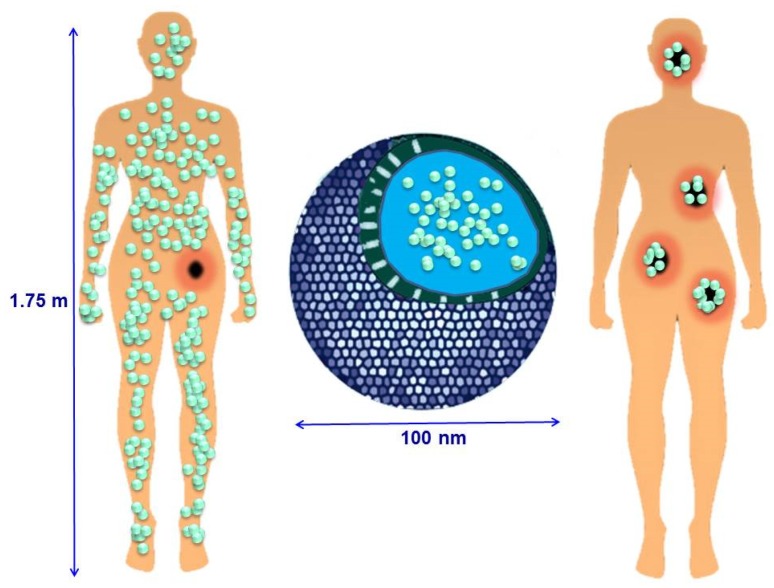
Schematic depiction of drug administration for cancer therapy: systemic treatments *versus* targeted therapies using nanomaterials.

In recent times, nanomaterials have been used more and more in healthcare, electronics, cosmetics and other areas [1,2,3]. Because of their small size, they have physical and chemical properties different from those of bulk materials and open up a new range of solutions for different problems, especially in nanomedicine [4,5,6,7,8,9,10]. The overall evolution of nanomaterials in medicine has provided more than 250 nanomedicine products that are approved or are in the course of different stages of clinical study [11]. Target-specific drug therapies and methods for early diagnosis of pathologies are the priority research areas in which nanotechnology plays a vital role. For instance, the development of nanotechnology has led to the development of powerful new nanodevices for early diagnosis, prediction, prevention and personalized treatment of cancer tumors [12,13,14,15]. According to the World Health Organization, cancer accounted for 8.2 million deaths in 2012, being 21.7 million the number of new cancer cases expected to be diagnosed, while 13 million cancer deaths are predicted in 2030 [16]. These figures make cancer one of the leading causes of death worldwide. As far as cancer therapeutics is concerned, the most common cancer treatments are restricted to chemotherapy, radiation and surgery, involving a lot of side effects caused by a non-specific tissue distribution of anticancer agents, insufficient drug concentrations at the tumor, unmanageable toxicity, limited possibility to get information about therapeutic responses and the development of multiple drug resistance due to the repeated exposition to chemotherapeutic agents inefficient drug concentrations reaching the tumor site, intolerable cytotoxicity, limited ability to monitor therapeutic responses and development of multiple drug resistance acquired upon repeated chemotherapeutic cycles [17,18,19]. The use of nanoparticles (NPs) is a promising alternative as they can hold, carry, protect and deliver therapeutic compounds specifically in the diseased tissue. NPs allow more effective and patient friendly treatment regimens by reducing drug concentration and dosing frequency, by offering easier administrations and by improving safety [20,21,22] (Figure 1).

**Figure 2:**
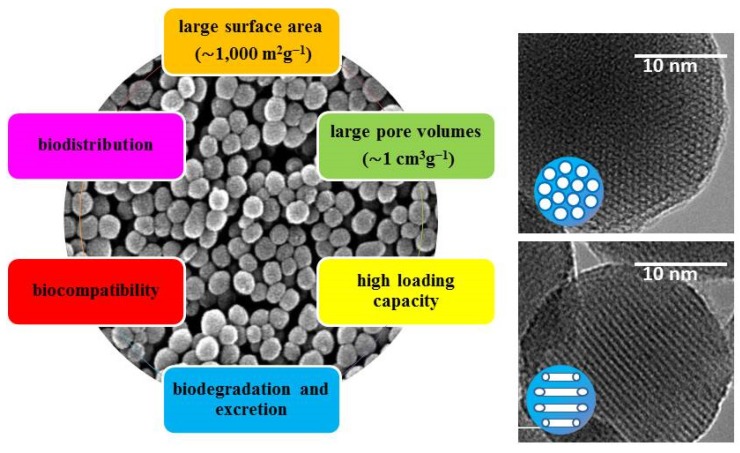
**Left**: Main characteristics of MSNs. **Right**: transmission electron microscopy (TEM) images of 2D-hexagonal MCM-41 type mesoporous silica nanoparticles (MSNs) taken with the electron beam parallel (**up**) and perpendicular (**down**) to the mesoporous channels.

Inorganic NPs show remarkable advantages compared to the organic ones, including high thermal, chemical and mechanical stability under physiological conditions and good biocompatibility. However, the main reason that makes NPs ideal candidates for the treatment of cancer is their ability to target and selectively accumulate in tumor tissue and release their cargo in a controlled manner once there. Among inorganic nanomaterials, mesoporous silica NPs (MSNs) are one of the most promising drug carriers, and they have attracted increasing attention in fields such as drug delivery, diagnostic and medical imaging and engineering due their unique properties (Figure 2) including: large surface area (~1000 m^2^·g^−1^ for MCM-41 type particles) and large pore volumes (~1 cm^3^·g^−1^) providing high loading capacity, high degree of tunability regarding size, morphology and pore diameter, biocompatibility, biodistribution, biodegradation and excretion [23,24,25,26,27,28,29,30,31,32,33,34,35,36,37,38,39,40]. Another added advantage is the ease of synthesis showing a great variety of morphologies and surface functionalities using different strategies which have been reviewed elsewhere [41,42,43,44,45,46].

The synthesis of MSNs can be carried out by using two different approaches. The first one is the so-called “modified Stöber method” [47], which consists in the condensation of silica under basic medium in the presence of cationic surfactants as structure directing agents. The second strategy is the aerosol-assisted synthesis, which allows using not only cationic but also anionic and non-ionic surfactants to obtain MSNs [48,49,50,51,52]. The surfactant removal usually leads to materials with cylindrical mesopores arranged in a two-dimensional hexagonal fashion, characteristic of MCM-41 type materials [53]. The resulting MSNs have an internal surface, *i.e.*, the inner part of the mesopores, and an external surface, *i.e.*, the external face of the NPs. This fact allows the selective functionalization of MSNs with different functional groups. Normally, the functionalization of MSNs is achieved either by co-condensation or by post-synthesis methods using organoalkoxysilanes, ((RO)_3_SiR’) [44,54]. This strategy allows incorporating different functionalities in MSNs for different purposes, such as promoting drug adsorption or covalently attaching fluorophores [23]. Usually, the fluorophore is pre-reacted with an aminosilane that is subsequently used in the cocondensation synthesis, yielding inherently fluorescent MSNs for cell imaging [55,56,57]. Indeed, when aimed at targeted smart drug delivery, additional moieties such as targeting agents, hydrophylic polymers, stimuli-responsive nanogates, *etc.* can be incorporated into MSNs to provide them with multi-functional properties.

In this manuscript, the recent developments on MSNs as stimuli-responsive drug delivery systems able to release therapeutic compounds once the target diseased tissues and cells are reached are overviewed.

## 2. Selective Targeting

Normal cells rely on the integrity of regulatory circuits that control cell proliferation and maintenance. The regulatory circuits are disrupted in cancer cells, and the type and behavior of the cancer cell vary depending on the type of damage caused to the regulatory circuits [58]. The abnormal behavior of cancer cells results in an excessive and rapid growth of solid tumors. This particularity can be exploited against tumor cells in attempts to treat the disease, using either passive targeting or active targeting, or a combination of both [35,36,59,60,61,62]. The use of targeted MSNs can also solve the lack of selectivity of some drugs since they can host, transport and guide the therapeutic agents selectively to the tumor. This strategy permits decreasing the high doses of cytotoxic drugs, which are needed in conventional chemotherapy, increasing therapeutic efficacy and diminishing undesired side effects.

### 2.1. Passive Targeting

**Figure 3:**
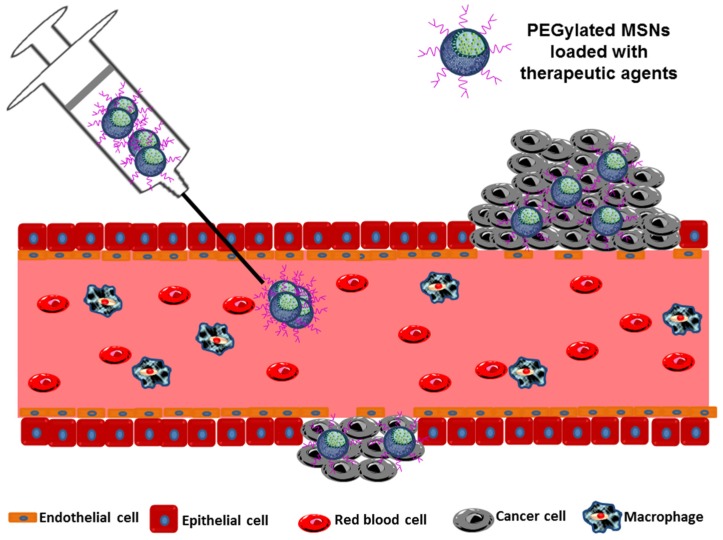
Schematic illustration of enhanced permeation and retention (EPR) effect.

It is known that solid tumors undergo an excessive and rapid growth which is associated with a high demand of nutrients and oxygen supply. As a consequence, some of them acquire the ability to promote the formation of new blood vessels from the surrounding capillaries; this process is termed as angiogenesis. However, this process is so fast and uncontrolled that the new vessels are irregular, exhibiting discontinuous epithelium and presenting fenestrations that can reach sizes in the 200–2000 nm range. When blood components reach tumor vessels, they extravasate throughout the fenestrations to the tumor interstitium. Besides, unlike normal tissues, in solid tumors the lymphatic drainage is deficient. Therefore, whereas molecules smaller than 4 nm can diffuse back to the bloodstream, the diffusion of NPs is impeded by their hydrodynamic radii and they accumulate in the tumor interstitium. This phenomenon is called “enhanced permeability and retention (EPR) effect” and was discovered by Matsumura and Maeda in 1986 when they realized that macromolecules greater than 50 kDa could preferentially distribute to the tumor interstitium and remain there for extended time periods [63] (Figure 3).

Nonetheless, accumulation of NPs in tumors will only take place if they avoid clearance by mechanisms such as renal clearance and uptake by the reticuloendothelial system (RES). Particle circulation time and the ability to overcome biological barriers are essential to attain successful passive targeting. Focusing on MSNs, three main features of these NPs affect these phenomena: (i) particle size; (ii) particle shape; and (iii) surface properties.

(i) *Particle size* is considered one of the most important features of NPs. MSNs must be at least 50 nm in diameter to keep their inherent mesoporosity and avoid renal clearance, but have to be smaller than 300 nm to diffuse through the tumor interstitium in sufficient amounts to achieve therapeutic effect [11,64].
(ii) *Particle shape* has gained considerable attention since it was found that non-spherical NPs could reduce phagocytosis by macrophages, thus exhibiting longer *in vivo* circulation times [65,66]. However, it is difficult to consider shape as a single variable because the fabrication techniques used to produce NPs with different shapes using biocompatible materials are limited [67]. Thus, it is difficult to exclude the relationship of various chemical, electrostatic and morphological factors by control experiments. In fact, there are only few reports regarding the effect of particle shape on *in vitro* and *in vivo* behavior, [68,69] even some of them with contradicting results. All these aspects make the influence of the shape of MSNs on cell internalization and cellular fate an unsolved question.
(iii) *Surface properties* are considered, together with the most important aspects that influence the EPR effect as it is the nature of the surface the first aspect that the MSNs “show” to the cells. Surface modification is one of the fundamental approaches used to increase the time MSNs remain in circulation to ensure tumor accumulation. The aim is to make MSNs “invisible” for the RES avoiding a rapid clearance. Surface functionalization with hydrophilic polymers is one of the most used strategies, especially with PEG (polyethylene glycol) and their derivatives (Figure 3) [70,71,72,73]. PEG not only reduces RES uptake but also improves the stability of the MSNs in biological fluids [71].

### 2.2. Active Targeting

As previously stated, solid tumor experience an uncontrolled rapid growth that requires increased nutrient supply, which makes diseased tissues and cells overexpressing different surface receptors. Usually, solid tumors are composed by a heterogeneous mixture of both cancer and healthy cells, and developing nanocarriers able to discriminate between them becomes an essential milestone. Thus, the basis of active targeting relies on “decorating” the periphery of the nanocarriers with targeting ligands which are specifically selected for a given receptor overexpressed in the surface of cancer cells or vasculature and poorly expressed in healthy cells or vessels.

**Figure 4:**
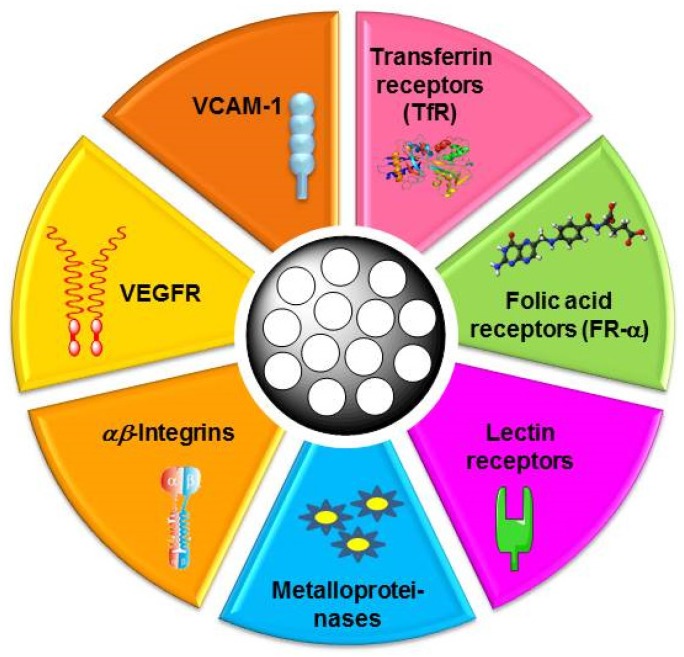
Molecular targets for active targeting of cancer by mesoporous silica nanoparticles: (i) tumor cell membrane receptors, such as transferrin receptors (TfR), folic acid receptors (FR-α) and lectin receptors; (ii) tumor vasculature receptors, such metalloproteinases, as αβ-integrins and vascular endothelial growth factor receptor (VEGFR). Molecular targets for active targeting of cancer by mesoporous silica nanoparticles.

The different molecular targets for active targeting of cancer by MSNs are schematically displayed in Figure 4. These molecular targets can be divided into two main groups: receptors overexpressed in the surface of cancer cells; and receptors overexpressed in the blood vessels supplying tumor tissue. Table 1 summarizes some of the targeting ligands that have been conjugated to MSNs to promote specific and selective recognition by tumor tissues. Different conjugation strategies have been developed to graft targeting ligands to MSNs, such as carbodiimide-mediated COOH/NH_2_ coupling, maleimide/SH coupling, *etc.* For further information about these issues, the reader is encouraged to consult references included in Table 1.

**Table 1:** Active targeting strategies for mesoporous silica nanoparticles.

Targeting Cell Membrane Receptors				
Receptor ^a^	Targeting Ligand ^b^	Conjugation Strategy ^c^	Target Cell Line ^d^	Ref.
TfR	Tf	CS-1	PANC-1, BT-549	[74]
TfR	Tf	CS-2	HeLa	[75]
TfR	Tf	CS-2	HT1080	[76]
EGFR	EGF	CS-3	HuH-7	[77]
FAR (FR-α)	FA	CS-2	Hela, PANC, U2Os, MDA-MB-231, SK-BR-3, MiaPaca-2	[78,79,80,81,82,83,84,85]
FR-α	Methotrexate	CS-2	HeLa	[86]
Sigma receptor	Anisamide	CS-2	ASPC-1	[85]
Importing α and β receptors	TAT peptides	CS-2	Hela; MCF-7/ADR	[87,88,89]
IL-13Rα2	IL-13 peptide	CS-3	U251	[90]
**Targeting Tumor Vasculature Receptors**				
**Receptor**	**Targeting Ligand**	**Conjugation Strategy**	**Target Cell Line**	**Ref.**
HER2	Anti-herceptin	CS-2	SK-BR3	[91]
HER2/neu	Anti-HER2/neu	CS-3	BT474	[92]
ErbB2	Anti-ErbB2	CS-4	MCF-7	[93]
Mesothelin	Anti-ME1	CS-2	MM	[94]
CD105/endoglin	Anti-TRC105	CS-3	HUVECs	[95]
NET	MABG	CS-2	NB1691-luc	[96]
α_ν_β_3_-integrins	c(RGDyK)	CS-3	U87-MG	[97]
α_ν_β_3_-integrins	cRGD	CS-5	MDA-MB 435	[74]
α_ν_β_3_-integrins	K7RGD, c-RGDFK	CS-2	HeLa	[98]
α_ν_β_3_-integrins	K_8_(RGD)_2_	CS-4	U87-MG	[99]
α_ν_β_3_-integrins	N_3_GPLGRGRGDK-Ad	CS-6	SCC-7, HT-29	[100]
α_ν_β_3_-integrins	N_3_RGDFFFFC	CS-5	U87-MG	[101]
α_ν_β_3_-integrins	Thiolated-RGD	CS-3	A375, HepG2, MCF-7, Neuro-2a	[102]
(VCAM-1)R	Anti-(VCAM-1)	CS-2	HUVEC-CS	[103]
VEGFR	VEGF	CS-3	U87-MG	[104]

^a^ TfR: Transferrin receptor; EGFR: Epidermal growth factor receptor; FAR (FR-α): Folic acid receptor; IL-13Rα2: Interleukin-13 receptor subunit alpha-2; HER2: epidermal growth factor receptor; ErbB2: Receptor tyrosine-protein kinase 2; NET: Norepinephrine transporter; (VCAM-1)R: vascular cell adhesion molecule 1 receptor; VEGFR: Vascular endothelial growth factor receptor; ^b^ Tf: Transferrin; FA: Folic acid; EGFR: Epidermal growth factor; TAT: Transactivator of transcription; IL-13: Interleukin-13; MABG: metaaminobenzyl guanidine (meta-iodobenzylguanidine analogue); c(RGD): Cyclic RGD (Arg-Gly-Asp); c(RGDyK): Cyclo (Arg-Gly-Asp-D-Phe-Lys); K_7_RGD: linear RGD peptide sequence with 7 consecutive lysine residues; K_8_(RGD)_2_: cationic peptide containing 2 RGD sequences; VCAM-1: vascular cell adhesion molecule 1; VEGFR: Vascular endothelial growth factor; ^c^ CS: Conjugation Strategy; CS-1: Epoxy/NH_2_ coupling; CS-2: COOH/NH_2_ carbodiimide-mediated coupling; CS-3: Maleimide/SH-mediated coupling; CS-4: Electrostatic interactions; CS-5: Disulfide exchange and S-S bond formation; CS-6: Ad/b-CD host-guest interaction; ^d^ PANC-1: Human pancreatic carcinoma, epithelial-like cell line; BT-549: Human breast carcinoma cell line; HeLa: Human epithelial cells from a fatal cervical carcinoma; HT1080: Fibrosarcoma cell line; HuH-7: Human hepatoma cell line; U20S: Human osteosarcoma cell line; MDA-MB 231 and 435: Human breast carcinoma cell lines; SK-BR-3: Human breast adenocarcinoma cell line; MiaPaca-2: Human pancreatic carcinoma cell line; ASPC-1: Human pancreas adenocarcinoma cell line; MCF-7/ADR: (ADR)-selected human breast cancer cell line; U251: glioma cell line; BT474: Human breast cancer cell line; MM: Multiple myeloma cell line; HUVEC: Human umbilical vein endothelial cell line; U87-MG: Human primary glioblastoma cell line; SCC-7: Squamous cell carcinoma; HT-29: Human intestinal epithelial cells; A375: Human amelanotic melanoma cell line; HepG2: Human hepatoblastoma-derived cell line; Neuro-2a: Mouse neuroblastoma cell line; HUVEC: Human umbilical vein endothelial cell line.

The first strategy to provide NPs with active targeting ability consists in decorating their outermost surface with certain ligands (antibodies, proteins, peptides, aptamers, saccharides or small molecules such as folic acid) able to be specifically recognized by surface receptors overexpressed in tumor cells. Thus, these targeted-MSNs could be specifically internalized by tumor cells by receptor-mediated endocytosis without affecting neighboring healthy cells (Table 1). For further information about the targeting receptor-mediated endocytotic pathways with NPs, readers are encouraged to read an interesting recent review of this topic [105]. This strategy is complementary to passive targeting via EPR effect to improve the efficiency of cancer therapy and decrease the side effects of conventional chemotherapy. Some common tumor cell membrane receptors include:

- *Transferrin receptor (TfR)*: Tf is a membrane glycoprotein that operates together with its receptor, TfR, to assist the uptake of iron by the cell. The TfR may be overexpressed by up to 100-fold on tumor cells, making it an attractive alternative for targeted delivery of drugs by grafting Tf to MSNs.
- *Folic acid receptor (FAR)*: FAR is one of the most widely studied molecules for targeting MSNs to cancer cells, since *FAR* is up-regulated in several types of human cancers, including ovarian, endometrial, colorectal, breast, lung, renal carcinoma, brain metastases derived from epithelial cancer and neuroendocrine carcinoma [106].
- *Epidermal growth factor receptor (EGFR)*: is a receptor tyrosine kinase that belongs to the ErbB family, which is extremely activated in many epithelial tumors. The receptor’s aberrant abnormal activation found in cancer can obey different mechanisms, including receptor overexpression, mutation, ligand-dependent receptor dimerization, and ligand-independent activation is a receptor tyrosine kinase of the ErbB family that is abnormally activated in many epithelial tumors. Several mechanisms lead to the receptor’s aberrant activation that is observed in cancer, including receptor overexpression, mutation, ligand-dependent receptor dimerization, and ligand-independent activation. Thus, targeting of NPs to EGFR by grafting EGF or anti-EGFR agents is a good alternative for cancer treatment [107].
- *Antigens*: Abnormal expression of certain antigens in the surface of tumor cells is the fundamental of antibody (*Ab*)-based cancer therapies [108]. The presence of cell surface antigens expressed by human cancers has provided a wide range of targets that are either overexpressed, mutated or selectively expressed in comparison definition of cell surface antigens that are expressed by human cancers has revealed a broad array of targets that are overexpressed, mutated or selectively expressed compared with normal tissues. This strategy can be used to target NPs to cancer cells. Thus, different *Abs* have been grafted to MSNs to design effective tumor-targeted nanodevices.

The second approach consists in targeting the blood vessels that irrigate solid tumor. During the formation of new blood vessels, tumor mass secrets different growth factors that promote angiogenic processes. The direct targeting of MSNs to the tumor vasculature disturbs nutrients and oxygen supply to the tumor mass and triggers its destruction. This strategy presents several advantages:

(i) Extravasation of NPs from the blood vessels is not required.
(ii) Tumor blood vessels usually overexpress certain receptors which are easily accessible to NPs.
(iii) Endothelial cells that compose the tumor vessels are less susceptible to suffer mutations than tumor cells, which reduces the risk of multidrug resistance. This fact obeys the more stable environment of endothelial cells compared to that of tumor cells housed inside the solid tumor mass, which are exposed to hard conditions (low pH values, low O_2_ pressure, *etc.*).
(iv) Endothelial cell markers are common in different tumors.

Some angiogenic markers that can be used as molecular targets include:

- *Vascular endothelial growth factor receptor (VEGFR)*: VEGF is a signal protein produced by cells to stimulate vasculogenesis and angiogenesis. The endothelial cells surrounding tumor cells overexpress VEGFRs. Thus, it is possible to target NPs to tumor blood vessels by grafting VEGF.
- *Vascular cell adhesion molecule-1 receptor ((VCAM-1)R)*: VCAM is a protein that mediates cell-to-cell adhesion. (VCAM-1)Rs are only expressed on the surface of tumor blood vessels and inflammation. The attachment of *Ab* specifically designed to bind to this molecule to NPs could be a good targeting strategy.
- *Matrix metalloproteinases (MMPs)*: are enzymes responsible of remodeling the extracellular matrix. These processes are necessary for a vast and varied array of physiological events (wound repair, organismal growth and development, and mediation of immune responses). MMPs degrade all kinds of extracellular matrix (ECM) proteins, playing a key role in angiogenesis and metastasis. Since MMPs are overexpressed in the extracellular environment of certain kinds of tumors, they can be used as a kind of tumor localization signal in cancer therapy [109,110]. Thus, some *Ab* able to selectively bind MMPs have been conjugated to different NPs, especially antibodies that recognize the membrane type-1 metallo-proteinase which is present on endothelial tumor cells of a large number of malignancies.
- αβ*-integrins*: are endothelial cell receptors for ECM proteins which are highly overexpressed in neovascular endothelial tumor cells but is scarcely present in healthy cells. Oligopeptides harboring the RGD sequence (Arg-Gly-Asp) bind selectively to this receptor.

## 3. Stimuli-Responsive Mesoporous Silica Nanoparticles

**Figure 5:**
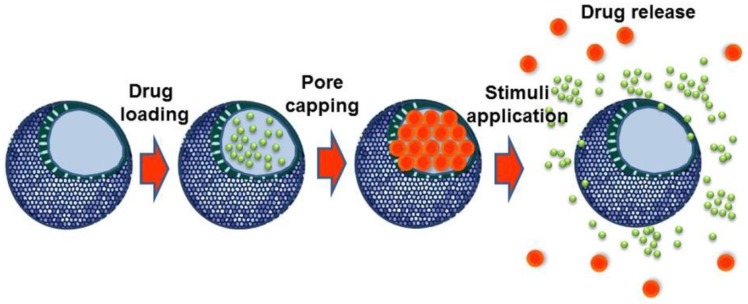
Schematic representation of the performance of stimuli-responsive MSNs.

Much research effort is being dedicated to develop novel stimuli-responsive nanomaterials able to release antitumor drugs once in the target tissue. Among nanomaterials, MSNs are of foremost interest due to their unique features and capability to are particularly interesting because of their unique characteristics and abilities to efficiently entrap, protect and specifically transport cargo molecules to tumor tissues [35,36,37,111]. In these smart nanosystems, the drug is enclosed within the mesopores and its output is blocked by using capping agents or gatekeepers that prevent any premature cargo departure. Drug release takes place once the system has been exposed to a given stimulus, which provokes the gatekeepers removal and triggers the release of the entrapped cargo. The release is triggered only upon exposure to stimuli, which induce the removal of gatekeepers and then the release of the entrapped drug molecules (Figure 5).

Table 2 summarizes representative stimuli-responsive drug delivery MSNs, which have been classified in two great groups depending on the stimulus that acts as release trigger, namely internal and external stimuli. The responsive linker and the capping agent are also indicated. In this section, we overview the recent advances in the development stimuli-responsive MSNs, discussing their advantages and constraints considering their potential clinical application.

**Table 2:** Stimuli-responsive strategies for smart drug delivery mesoporous silica nanoparticles.

	Stimuli	Responsive Linker	Capping Agent	Ref.
External	Temperature	Octadecyl (C_18_) chains	Paraffins	[112]
	Temperature	PNIPAm	PNIPAm	[113]
	Temperature	DNA strands	Biotin	[114]
	Temperature	Coiled-coil peptide motifs	Coiled-coil peptide motifs	[115]
	Electric field	4(3-cyanophenyl)butylene dipolar molecule	-	[116]
	Magnetic field	Hybridization of 2 ssDNA	γ-Fe_2_O_3_ NPs	[117]
	Magnetic field	Alkylamonium chains (NH_3_^+^–(CH_2_)–NH_2_^+^–R)	CB[6]	[118]
	Magnetic field	PEI/PNIPAM polymer	PEI/PNIPAM chains + catalase	[119]
	Magnetic field	Azo bonds (–N=N–)	PEG	[120]
	Light	4-[4-(1-(Fmoc)methyl)-2-methoxy-5-nitrophenoxy]butanoic acid photolinker	Protein shell (avidin-estreptavidin-biotin-transferrin)	[76]
	Light	DNA aptamer	DNA aptamer	[121]
	Light	Azobenzene/coumarin dimer	Coumarin dimer	[122]
	Light	Azobenzene derivatives	β-CDs	[123]
Internal	pH	Acetal linker	Au NPs	[124]
	pH	Boronate ester	Fe_3_O_4_ NPs	[125]
	pH	Ferrocenyl moieties	β-CD-modified CeO_2_ NPs	[126]
	pH	PAH-PSS PEM	PAH-PSS PEM	[127]
	pH	Aromatic amines	CDs	[128]
	pH	Benzoic-imine bonds	Polypseudorotaxanes	[129]
	pH	CaP soluble at acid pH	CaP coating	[130]
	Redox potential	–S–S–	ssDNA	[131]
	Redox potential	–S–S–	PEG	[132]
	Redox potential	–S–S–	CdS NPs	[133]
	Redox potential	–S–S–	PPI dendrimer	[134]
	Enzymes	MMP-degradable gelatin	Gelatin coating	
	Enzymes	β-galactosidase-cleavable oligosaccharide	β-galacto-oligosaccharide	[135]
	Enzymes	MMP9-sensitive peptide sequence (RSWMGLP)	Avidin	[136]
	Enzymes	Protease-sensitive peptide sequences (CGPQGIWGQGCR)	PNIPAm-PEGDA shell	[137]
	Enzymes	α-amylase and lipase cleavable stalks	CDs	[138]
	Enzymes	HRP-polymer nanocapsule	-	[139]
	Enzymes	Phosphate-phosphate APasa -hydrolizable bonds	ATP	[140]
	Small molecules	Ionizable benzimidazole group	CD-modified glucose oxidase	[141]
	Small molecules	*pAb*	*pAb*	[142]
	Small molecules	ATP aptamer	ATP aptamer	[143]

PNIPAm: Poly(*N*-isopropylacrylamide); ssDNA: single-stranded DNA; CB[6]: Cucurbit[6]uril; PEI: poly(propylene imine); PEG: poly(ethylneglycol); CD: cyclodextrin; PAH: poly (allylamine hydrochloride); PSS: sodium poly(styrene sulfonate); PEM: polyelectrolyte multilayers; APase: acid phosphatase; PEGDA: poly(ethylene glycol) diacrylate; HRP: enzyme horseradish peroxidase; ATP: adenosine triphosphate; *pAb:* polyclonal antibody; MMP: matrix metalloproteinase.

### 3.1. External Stimuli

In this section, we focus on MSNs’ drug delivery systems that respond to external stimuli, such as temperature, electric fields, magnetic fields and light. In this case, an apparatus is needed to trigger drug release, which allows remotely controlling drug release and in some cases operating via “on-off” switching mechanisms.

#### 3.1.1. Temperature

Researchers have strived to construct thermal-responsive MSNs for clinical applications. This approach permits an accurate and local control of drug release in unhealthy organs or tissues [144]. It is possible to artificially increase the temperature using external heat sources, heated fluid or magnetically induced hyperthermia, for instance. Grafting temperature-sensitive gatekeepers on MSNs’ surface allows controlling drug release applying temperature gradients. Thermolysis relies on a thermal stimulus to trigger cleavage of the chemical bond that constitutes the sensitive-linker, such as an Au–S or diazo (–N≡N–) bonds [145,146]. However, most of the temperature-responsive drug delivery systems consist in combining thermosensitive polymers with MSNs (Table 2). The most widely used polymers are those that are hydrophilic below their lower critical solution temperature (LCST). When the temperature rises above LCST, the polymer becomes hydrophobic and its conformation changes from an expanded or “coil” (soluble) to a contracted or “globular” (insoluble) state [147]. Despite it is a relatively simple drug delivery mechanism, *in vivo* application still remains a challenge due to the lack of effective methods to localize heat exposure only to diseased tissues. Nonetheless, this issue has been overcome in part by integration magnetic NPs into MSNs containing thermosensitive moieties. Magnetic NPs are able to generate a localized thermal effect upon exposure to an alternating magnetic field (AMF), which permits triggering drug release from thermosensitive MSNs-based systems [116,117,118,119].

#### 3.1.2. Electric Field

Electric fields are widely used as power and signal sources relying on the fact that electrotherapy has been developed to cure various diseases, such as brain diseases, voice and swallowing disorders, chronic resistant wounds, tumors, *etc.* [148,149,150]. However, studies on electric field-responsive MSNs are quite limited despite the achievements involving electrochemical processes [151,152]. Unlike other stimuli that require the use of large or specialized equipment, electrical signals are easy to generate and control [153]. For instance, it is possible to assemble functional molecules with permanent electric dipole moments into mesoporous channels (Table 2) [116]. Upon application of an electric field, the flexible molecular chains swing to push the guest molecules out of the pore voids.

#### 3.1.3. Magnetic Field

Magnetic fields have also attracted much attention since they play crucial roles in biomedical applications such as therapy and imaging. The use of the magnetic field as a release trigger is related to temperature, as above mentioned, and relies on the integration of magnetic NPs in MSNs systems to generate heat upon remote application of an AMF. Among magnetic NPs, superparamagnetic iron oxide NPs are mostly used [3,30,154]. These particles are the most suitable for biomedical applications because their remaining magnetization when they are suspended in biological fluids ceases on removal of the external AMF, showing lower aggregation drawbacks than other magnetic NPs. Some examples of magnetic field-triggered drug release from MSNs are displayed in Table 2.

#### 3.1.4. Light

**Figure 6:**
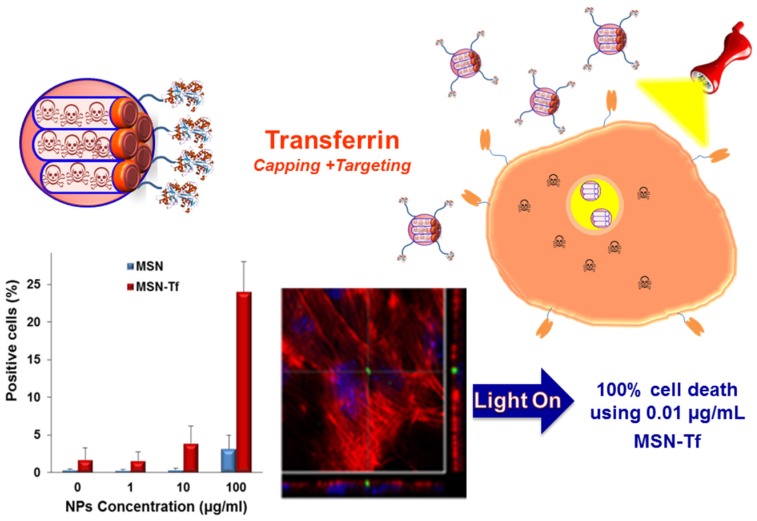
**Up**: Schematic illustration of the action mechanism of light-responsive nanosystem based in MSNs decorated with a biocompatible protein shell (transferrin, Tf, grafted to MSNs using a light cleavable photolinker), affording MSN-Tf. **Down**: Cellular uptake of MSNs and MSN-Tf labeled with fluorescein. Confocal microscopy images show NPs (green) inside tumor cells (actin in red, nucleus in blue). The light irradiation of MSN-Tf provokes the cleaving of the photolinker, which triggers pore uncapping and subsequent drug release [76].

Light-operating nanodevices have attracted special interest for their controllability and rapid-responsiveness [155,156]. Light as external stimulus to trigger drug release from MSNs has many advantages, including remote responsiveness, non-invasiveness, highly controllable properties, low toxicity and convenient operation without affecting the neighboring zones. Light-responsive switches usually involve photoisomerization, photodimerization or photocleavage. One of the most exploited strategies consists in grafting MSNs with light-sensitive linkers capable of undergoing physicochemical changes (photoisomerization, photodimerization, *etc.*) or cleavage upon light irradiation (Table 2). Recently, an innovative light-responsive nanosystem consisting of MSNs decorated with a biocompatible protein shell cleavable by light irradiation has been reported (Figure 6) [76]. The proteins that compose the protein shell (avidin, streptavidin and biotinylated transferrin) plays a dual role acting both as targeting and capping agent, which avoid the use of redundant systems. The light responsive behavior is provided by a biotinylated photolinker covalently grafted to the mesoporous surface, which suffers photocleavage upon UV radiation (366 nm) and permits the release of the entrapped drug. The cytotoxic capacity of this system was evaluated *in vitro* against HT1080 cellular line which overexpresses transferrin receptors (TfR), showing an excellent performance being able to destroy the diseased cells using a very low particle dose (100% cell using 0.01 µg/mL of the nanosystem).

### 3.2. Internal Stimuli

In this section, we discuss the recent developments in MSNs able to response to internal stimuli, *i.e.*, those sensitive to chemical variations that arise in certain sites inside the human body. Smart MSNs that respond to internal stimuli have the advantage of not being invasive, since they do not require external mediation to trigger drug release. However, the control over drug dosage is lower compared to release nanosystems operating under external stimuli. Herein, we focus on drug delivery from MSNs triggered by internal stimuli, including pH modifications, variations in the redox potential and presence of certain enzymes or small molecules.

#### 3.2.1. pH

pH is one of the most exploited internal stimulus to trigger drug release from nanomedicines and it has become the focus of numerous investigations in oncology [157]. The cancer process evolves with pH values different than those of healthy conditions. Thus, the extracellular pH of normal tissues and blood is approximately 7.4, whereas that in a tumor microenvironment is between 6.0 and 7.0, which is mainly caused by high glycolysis rate and high level of CO_2_ [61]. Moreover, when a NP is internalized inside the cell, it is exposed to different pH depending on the cell compartment or organelle [158]. Therefore, the pH value will drop further from the extracellular microenvironment of a tumor to intracellular organelles, such as endosomes (pH = 5.5) and lysosomes (pH < 5.5). Therefore, the abnormal pH gradients combined with the advantages of MSNs provide opportunities to develop pH-responsive MSNs as drug delivery systems for cancer treatment. Many research groups have reported on pH-responsive MSNs modified with various gatekeepers. The triggered release of anti-cancer drugs from mesoporous channels has mainly been achieved by using polyelectrolytes; pH sensitive polymers such as poly(4-vinylpyridine), poly(2-(diethylamino)ethyl methacrylate), chitosan, starch or poly(styrene sulfonate); supramolecular nanovalves; pH-sensitive linkers (boronate, acetals, hydrazone, *etc.*); and acid-decomposable inorganic materials, among others. Some examples are shown in Table 2. Finally, an alternative strategy that does not require the use of pore capping agents consists in directly grafting the cytotoxic drugs to the surface of MSNs using pH-sensitive linkers [159,160].

#### 3.2.2. Redox Potential

Another interesting approach to developing smart drug delivery nanodevices is to take advantage of the different concentrations of certain reductive species, such as glutathione (GSH), between the intra-cellular and the extra-cellular space and also between normal and tumor tissues [161,162]. For instance, inside the cell, there are 1000 times more GSH than in the extracellular media. As GSH is able to cleave disulfide (S–S) groups, different capping agents, such as inorganic NPs, organic molecules and polymers, have been grafted to MSNs via disulfide bonds. Some examples are displayed in Table 2. Once the nanosystems are internalized and reach the intracellular space, the presence of increased amounts of GSH triggers the rupture of disulfide bonds, which leads to mesopore opening and permits drug release. Another reported strategy consists in the direct immobilization of the therapeutic agent into MSNs using GSH-cleavable disulfide bonds [163,164].

#### 3.2.3. Enzymes

Nanomaterial uptake occurs primarily via receptor-mediated endocytosis in which nanomaterials are taken up into the cytosol through vesicles that finally are being fused with lysosomes [162]. The interiors of such cellular vesicular compartments contain numerous enzymes, especially acid hydrolases [165,166,167]. Enzyme responsive ensembles are especially appealing to prepare tailor-made nanodevices due to the high selectivity, biological stability, function under mild conditions and efficient catalytic capability of enzymes, which paves the way to construct MSNs-based triggered release systems with the highest specificity, accuracy and efficiency [168]. Besides, there are other enzymes which are overexpressed in certain tumors [169,170,171], which can be also exploited as release triggers. Some representative examples of smart nanodevices consisting of MSNs end-capped via enzyme-cleavable linkages are displayed in Table 2.

**Figure 7:**
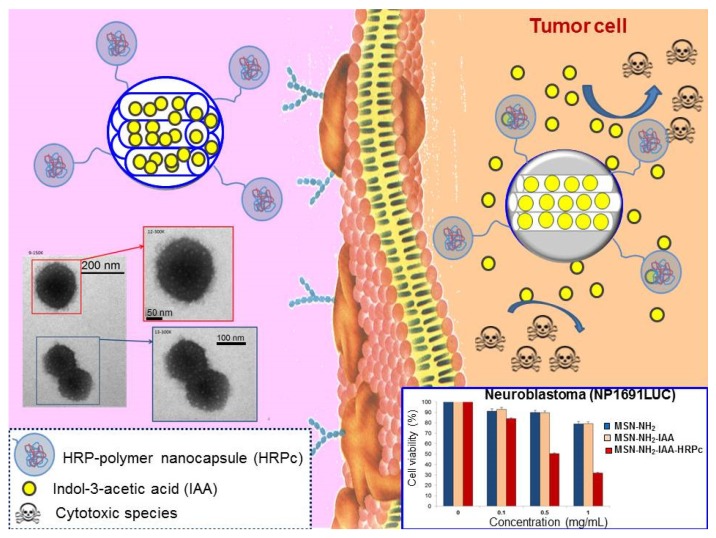
Schematic illustration of the *in situ* cytotoxic generation for antitumor therapy [139]. (i) Functionalization of MSNs with amino group (MSN-NH_2_); (ii) loading of the pro-drug indol-3-acetic acid (IAA) (MSN-NH_2_-IAA); grafting of an enzyme horseradish peroxidase (HRP)-polymer nanocapsule to the external surface of the nanosystem (MSN-NH_2_-IAA-HRPc). TEM images of the nanosystem and cytotoxicity studies with neuroblastoma cells are also displayed.

Very recently, a sophisticated approach consisting in the incorporation of enzymes in the nanosystem itself has been reported [139]. This strategy permits overcoming the limitation that, in some cases, is presented by the scarce concentration of the activating enzyme in the target zone, which is not sufficient to provoke a significant response. In this case, the enzyme responsible for the drug activation was covalently anchored on the external surface of MSNs. The enzyme wast firstly covered with a protective polymeric shell that permits the grafting to the silica surface was previously coated with a protective polymeric shell that allows the attachment on the silica surface while preserving its activity (Figure 7). In this work, indol-3-acetic acid (IAA), which was selected as pro-drug, was loaded into the mesopores and the enzyme horseradish peroxidase (HRP) coated with a protective polymeric shell was grafted to the external surface of MSNs. Once MSNs are internalized by tumor cells, intracellular enzymes degrade the protective polymeric shell. This permits HRP to oxidize IAA molecules, which produces cytotoxic substances, mainly reactive oxygen species (ROS) capable to destroy human cells by damaging membrane and DNA compounds, mainly hydroxyl and reactive oxygen species (ROS), able to destroy human cancer cells by membrane and DNA damage [172]. The efficacy of this novel nanosystem for antitumor purposes was *in vitro* demonstrated using neuroblastoma cells, which opens the gates for further *in vivo* studies for oncology therapy.

#### 3.2.4. Small Molecules

As in the previous case, some diseases are characterized by the production or accumulation of unbalanced amounts of certain chemical species. These agents can be employed as trigger events for drug delivery applications. Some examples of MSNs capped with gatekeepers sensitive to small molecules, such as glucose, antigens, adenosine triphosphate (ATP), *etc.*, are presented in Table 2.

### 3.3. Multi-Stimuli Responsive Mesoporous Silica Nanoparticles

As tumor formation is a complex and multifactorial process, the combination of several drug delivery systems increases the likelihood of activation and, thus, an increase of their effectiveness would be also expected. This can be achieved by combining several types of stimuli responsive MSNs or by multi-responsive controlled drug delivery systems. Thus, different smart MSNs capable of releasing their cargo triggered by two stimuli, such as pH and glucose, [173] pH and GSH [101,174], enzymes and temperature [175], pH and temperature [176], among others, have been developed.

Significant scientific effort has been committed to designing MSNs with pH and photoswitched drug release capability [177,178,179]. Within these systems, dual-stimuli MSNPs able to operate as AND logic gates are worth mentioning [178]. In this case, two different molecular machines were mounted on MSNs, namely, azobenzene as light-activated nanoimpellers and [2] pseudorotaxanes as pH-responsive nanovalves. Both molecular machines can act individually, but only the simultaneous activation of the two systems triggers cargo release. Recently, a dual pH-responsive MSN-based drug release system capable of respond both to the cancer extracellular and intracellular pH stimuli, has been described for synergistic chemo-photodynamic therapy [179]. By covalently linking histidine onto The two systems can act separately, but only the simultaneous activation of both molecular machines leads to cargo release. Very recently, an innovative dual pH-responsive MSN-based drug delivery system, which can respond to the cancer extracellular and intracellular pH stimuli, has been reported for synergistic chemo-photodynamic therapy [179]. By grafting histidine onto MSNs, the acid sensitive PEGylated cis-aconitic anhydridide tetraphenylporphyrin zinc (Zn-Por-CA-PEG) act as gatekeeper, blocking the nanopores by interaction between histidine and Zn-Por. Ath the extracellular pH of the tumor, *ca.* 6.8, the pH sensitive CA between PEG and Zn-Por breaks and the surface of Zn-Port contains positively charged amino groups to improve interaction between Zn-Por and histidine. At the cancer extracellular pH of *ca.* 6.8, the pH sensitive CA between Zn-Por and PEG cleaves and the surface of Zn-Por consists in amino positively charged to improve cell internalization. Besides, at the intracellular acidic microenvironment of *ca.* 5.3, the interaction between Zn-Por and hystidine is weakened, which provokes the removal of the gatekeeper and the Zn-Por departure. The photosensitivity of Zn-Por then allows combining chemotherapy and photodynamic therapy. This dual pH sensitive MSN-based drug delivery system provokekd higher *in vitro* cytotoxicity than the single chemotherapy of free DOX or photodynamic therapy of Zn-Por. This opens promising expectations in cancer therapy by overcoming the challenges in the efficient and specific drug release in the tumor site, and ideal antitumor efficacy. results in the removal of the gatekeeper and the Zn-Por drug release. The photosensitivity of Zn-Por further permits combining chemotherapy and photodynamic therapy. This dual pH sensitive MSN-based drug delivery system showed higher *in vitro* cytotoxicity than the single chemotherapy of free DOX or photodynamic therapy of Zn-Por, presenting its great potential for cancer treatment to overcome the challenges in efficient delivery in the site and ideal anti-cancer efficacy.

Finally, complex multi-responsive MSNs capable of releasing drugs in response to more than two stimuli have been also reported [180,181].

## 4. Safety, Tissue Accumulation and Elimination of Mesoporous Silica Nanoparticles

The safety of cancer therapies based on MSNs needs to be investigated according to *in vivo* protocols to evaluate their absorption, distribution, metabolism, excretion and toxicity [182,183]. As it is the case of pharmaceuticals, “the dose makes the poison”, and thus it is very important to define the concentration above which MSNs are no longer therapeutic but toxic. Toxicity must be identified for each type of MSNs after single (acute toxicity) and repeated (chronic toxicity) administration. Also, different exposure routes have to be compared [180]. Generally, silica-based materials are considered biocompatible and suitable for *in vivo* use [184,185,186]. MSNs, with the same composition to traditional silica NPs, exhibit characteristic features than may alter biological behaviors. For instance, MSNs provoked substantially lower hemolytic effect than non-porous silica NPs due to the decreased silanol groups accessible to the cell membranes of mammalian red blood cells in the former [187]. Albeit numerous *in vitro* studies have demonstrated the low cytotoxicity of MSNs against different cell lines, several reports have indicated that residual structure directing agents, mainly ionic surfactants, could provoke severe cytotoxicity when they are not completely removed from MSNs by traditional extraction methods [188,189,190].

Zink *et al.* investigated the maximum tolerated dose (MTD) of fluorescent MSNs via intravenous administration into female nude mice with the dosage ranging from 10 mg/kg to 200 mg/kg, once per day for 10 days [80]. The results indicated that all mice were generally healthy, but the mice treated with dosages higher than 100 mg/kg showed some liver enzyme alterations. Long-term toxicity evaluations using healthy nude mice with the dose of 1 mg/mouse per day indicated that there were not anomalous responses during a two month period. However, studies carried out on female CD-1 mice indicated that the MTD of MSNs was only 30 mg/kg [191]. Above MTD, the major affected organs were kidney and lungs. The toxicity was lessened by modifying MSNs with amine groups, which increased the MTD to 150 mg/kg. In another study, it was demonstrated that intraperitoneal or intravenous administration of 1.2 g/kg MSNs was lethal to SV 129 mice but was safe when reduced to 40 mg/kg [192]. For hollow MSNs, the lethal dose 50 of 110 nm rattle-type MSNs was higher than 1000 mg/kg after single dose administration, and no death was observed by repeated administration of dose of 80 mg/kg during 14 days [193].

MSNs targeted with given ligands can affect organs’ distribution and, hence, the safety profile of these nanosystems. Testing biocompatibility and safety of MSNs is mandatory due to the great variations of characteristics in this type of materials is demanding due to the immense variation of characteristics in this class of materials [194]. For optimal cancer therapy, MSNs should reach tumor tissues without provoking adverse effects in normal tissues. In this sense, the bio-behavior, highlighting *in vivo* biodistribution, of MSNs is strongly related to the preparation procedures [188,189], particle sizes [189], particle shape [69] and surface modification. As previously mentioned, PEGylation of MSNs substantially improves the blood compatibility (e.g., much lower hemolysis effect) and reduces the non-specific binding to serum proteins, which increases the half-life of MSNs most likely by escaping recognition by RES organs [195]. Several research groups have proved MSNs accumulation in liver and spleen after systemic administration [189,196,197].

Meng *et al.* investigated the biodistributions of MSNs of different particles sizes (80, 120, 200 and 360 nm) and their corresponding PEGylated counterparts (PEG-MSNs) [70]. As expected, the blood-circulation lifetime of PEG-MSNs was relatively longer. On the other hand, independently of the size, MSNs and PEG-MSNs mainly accumulated in liver and spleen after tail intravenous injection due to the recognition and phagocytosis of NPs by liver and spleen phagocytes, and few MSNs were found in the lungs and kidneys, and very few in the heart. Both MSNs and PEG-MSNs of larger particles sizes were more easily captured by the organs, which facilitates their degradation [103]. PEG-MSNs of smaller particle size escaped more easily from the capture by liver, spleen and lung tissues, exhibited longer blood-circulation lifetimes and were more slowly biodegraded and, consequently, had lower excretion rates [70].

There are only few reports regarding the effect of the shape of MSNs on their *in vitro* and *in vivo* behavior. Huang *et al.* designed MSNPs exhibiting similar particle diameter, chemical composition and surface charge but with different aspect ratios (ARs) (length: width) and evaluated their capability of being internalized by tumor cells via non-specific cellular uptake [68,69]. The results derived from *in vitro* tests indicated that particles with bigger AR (long rod shape) were taken up in larger amounts and showed faster internalization rate than particles with AR of 1 (spheres). The differences in the curvature of MSNs could be responsible for this different behavior. Thus, rod-shaped MSNs would have larger contact area with the cell membrane than spherical MSNs as the longitudinal axis of the rod interacts with the cell membrane. The influence of MSNs’ shape on the biodistribution, clearance and biocompatibility has been also investigated using MSNs with different ARs [69]. It was found that short-rod MSNs mainly accumulated in the liver, but long-rod MSNs were more easily trapped in the spleen. After PEGylation, the content of MSNs in the lung increased. The effect of MSNs’ shape on biocompatibility, such as hematology, serum chemistry and histopathology, was not apparent.

The above mentioned parameters together with dissolution kinetics affect blood circulation time and clearance. In this sense, dissolved silica is known to be adsorbed or excreted by the body [198]. There are several reports that support elimination of MSNs through renal and hepatic routes in the form of urines and feces containing either solid MSNs or degraded products, being the renal excretion the major route [69,189,199] Unexpectedly, given the renal cut limit of 5–6 nm, several reports demonstrated intact MSNs in the urine but the exact excretion process remains unclear [23,93].

## 5. Current Challenges of Mesoporous Silica Nanoparticles

Multidrug resistance (MDR) is the most important impediment for successful chemotherapy even with targeted drugs or/and combination chemotherapy [200]. In cancer chemotherapy, often drug-sensitive cells are killed, but a proportion of drug-resistant cells are left. The remaining cancer cells would grow again and result in tumor relapse and metastases. MSNs’ drug delivery systems can facilitate cellular uptake, increase intracellular accumulation and decrease cellular efflux in drug-resistant cancer cells. However, it is not sufficient for overcoming MDR. Another aspect to consider is the long and tortuous journey that MSNs experience *in vivo* since they enter the bloodstream until their intracellular action takes place [201,202]. MSNs must overcome stages of circulation, extravasation, accumulation, distribution, endocytosis, and endosomal escape before actuating. Most MSNs specialize in one or two of these stages, but each step is critical to the overall success of the therapy. This is why many MSNs probably get lost or spoiled on the way and do not reach their destination, or do it but in a non-functional state.

Another major requirement of MSNs for cancer therapy purposes is an enhanced penetration capability within the solid tumor. The current *in vitro* tests are carried out using bi-dimensional cell cultures, which do not provide any information about the penetration capacity in living tissues. Thus, it would be desirable to use more realistic *in vitro* models, such as tumor spheroids, *ex-ovo* chick embryo models or 3D cell cultures. Another issue that must be taken into account regards the colloidal stability of MSNs in physiological media. This is of foremost relevance in the case of targeted stimuli-responsive MSNs, whose production requires multiple synthetic steps which could lead to irreversible aggregation. In this sense, the colloidal stability of MSNs and also after storing conditions should be evaluated using media that mimics as much as possible the physiological conditions. This is essential to guarantee that the colloidal stability and the stimuli-responsive behavior are maintained in living tissues. This would allow for the industrial production and clinical translation of MSNs using cost-effective and scalable manufacture industrial production and clinical translocation of MSNs using cost-effective and scalable fabrication methods, which is still a great challenge [203].

The potential of active over passive tumor targeting in MSNs remains an unanswered question. As opposed to passive targeting, which mainly relies on the physiopathological properties of tumor tissue and circulation lifetime of MSNs, active targeting requires the incorporation of specific ligands. Promising *in vitro* results have been found, regarding improved binding, cellular uptake and efficacy of MSNs, but there is not clear evidence that the active targeting really improves their *in vivo* accumulation. In fact, there is a crucial concern about whether *in vitro* success of MSNs can also be reproduced *in vivo*. Generally, in the first decade of this century, researchers have focused on the basic characteristics of MSNs and their ability to deliver different kinds of anticancer drugs in cultured cells. Unfortunately, there are limited experimental data about *in vivo* fate of MSNs, which limits our knowledge about their clinical applicability in cancer therapy.

## 6. State of the Art

Since MSNs were proposed as drug delivery systems almost 20 years ago, they have undergone rapid development and have proven to be effective and versatile nanomaterials for the potential treatment of cancer and other diseases. Nowadays, it is possible to design multidrug nanodevices that can selectively target specific tumors and, once there, release their payload in response to certain stimuli. Despite the great progress of MSNs as antitumor nanomedicines, the biomedical application of these nanomaterials is only feasible if a deep understanding of their *in vivo* biocompatibility/toxicity and *in vivo* biodistribution is acquired. The versatility of MSNs highlights them as interesting drug delivery nanocarriers. Biological performance and applicability of MSNs have been demonstrated by preclinical experimentation, but systematic testing of biodistribution, safety and therapeutic efficacy by comparing various designs are still demanded to permit their translation from the lab bencch to and therapeutic efficacy as related to various designs are still needed to bring the technology closer to the clinic. Nowadays, our understanding of how nanoparticles behave in the human body is extremely limited, perhaps because it is not possible to achieve such encouraging results in clinical stages as in previous stages. However, our ability to manufacture particles with the desired characteristic will improve with time, as will our understanding of what characteristics will optimize efficacy against a given tumor.
